# Patient-reported outcomes, health-related quality of life, and acute medication use in patients with a ≥ 75% response to eptinezumab: subgroup pooled analysis of the PROMISE trials

**DOI:** 10.1186/s10194-022-01386-z

**Published:** 2022-02-07

**Authors:** Richard B. Lipton, Larry Charleston, Cristina Tassorelli, Thomas Brevig, Joe Hirman, Roger Cady

**Affiliations:** 1 Department of Neurology, Albert Einstein College of Medicine, Bronx, NY USA; 2 Department of Neurology, Michigan State University College of Human Medicine, East Lansing, MI USA; 3 Neurorehabilitation Unit, IRCCS Mondino Foundation, Pavia, Italy; 4 Department of Brain and Behavioral Sciences, University of Pavia, Pavia, Italy; 5 H. Lundbeck A/S, Copenhagen, Denmark; 6 Pacific Northwest Statistical Consulting, Woodinville, WA USA; 7 Lundbeck LLC, Deerfield, IL USA

**Keywords:** Eptinezumab, Responder analysis, CGRP monoclonal antibody

## Abstract

**Background:**

PROMISE-1 and PROMISE-2 evaluated the preventive efficacy, tolerability, and safety of eptinezumab, a calcitonin gene-related peptide–targeted monoclonal antibody, in adults with episodic (EM) and chronic migraine (CM), finding significant reductions in migraine frequency. This post hoc analysis compared patient-reported outcomes (PROs), health-related quality of life (HRQoL) and acute medication use in patients with a ≥ 75% migraine responder rate (MRR) after treatment with eptinezumab to patients with a ≥ 50– < 75% MRR.

**Methods:**

PROMISE-1 and PROMISE-2 were phase 3, randomized, double-blind, placebo-controlled studies. This analysis included patients from both studies treated with eptinezumab 100 mg or 300 mg who experienced ≥ 75% and ≥ 50–< 75% MRR over Weeks 1–12 (wks1–12). In both studies, HRQoL was measured by the 36-item Short-Form Health Survey (SF-36) and acute medication usage. PROMISE-2 also included the 6-item Headache Impact Test (HIT-6), patient-identified most bothersome symptom (PI-MBS), and Patient Global Impression of Change (PGIC).

**Results:**

In PROMISE-1, a total of 115/443 (26.0%; 100 mg, *n* = 49, 300 mg, *n* = 66) and 120/443 (27.0%; 100 mg, *n* = 61, 300 mg, *n* = 59) eptinezumab-treated patients achieved ≥ 75% and ≥ 50–< 75% MRR over wks1–12, respectively. In PROMISE-2, a total of 211/706 (30.0%; 100 mg, *n* = 95; 300 mg, *n* = 116) and 209/706 (29.6%; 100 mg, *n* = 110, 300 mg, *n* = 99) eptinezumab-treated patients achieved ≥ 75% and ≥ 50–< 75% MRR over wks1–12, respectively. EM and CM patients with ≥ 75% and ≥ 50–< 75% MRR over wks1–12 showed reduced use of acute headache medication and increased HRQoL to normative levels across SF-36 domains of bodily pain, social functioning, and physical functioning. In CM patients with ≥ 75% and ≥ 50–< 75% MRR over wks1–12, the mean change in HIT-6 total score with eptinezumab (pooled) was − 11.7 and − 7.6, respectively. “Very much” or “much” improvement responses were reported in 41.8% and 16.5% on PI-MBS and 36.2% and 20.0% on PGIC in ≥ 75% and ≥ 50–< 75% MRR, respectively.

**Conclusion:**

Eptinezumab treatment induced a ≥ 75% MRR over wks1–12 in the majority of patients. This patient subgroup reported substantial improvements in PROs associated with headache-related life impact and HRQoL, and reductions in acute headache medication use, which were more marked than those in the ≥ 50–< 75% responders. This study supports the clinical meaningfulness of ≥ 75% MRR for patients with either EM or CM.

**Trial registration:**

ClinicalTrials.gov identifiers: NCT02559895 (PROMISE-1), NCT02974153 (PROMISE-2).

**Supplementary Information:**

The online version contains supplementary material available at 10.1186/s10194-022-01386-z.

## Background

Eptinezumab is a monoclonal antibody against calcitonin gene-related peptide (CGRP) indicated for the preventive treatment of migraine in adults [1]. As a humanized immunoglobulin G1 (IgG1) antibody, eptinezumab rapidly and durably binds CGRP, thus providing sustained blockade of this key neuropeptide’s interaction with its receptor [2–4]. In the pivotal phase 3 PROMISE-1 and PROMISE-2 studies, eptinezumab 100 mg and 300 mg demonstrated rapid and sustained reductions in migraine frequency [5–8]. In both studies and at both dose levels, statistically significant reductions in mean monthly migraine days (MMDs) over Weeks 1–12, the primary efficacy endpoint, were achieved [5, 6]. Migraine preventive effects were observed early (> 50% reduction in migraine prevalence on the day following the initial dose versus the average in the screening period) and were sustained for the duration of the studies (PROMISE-1, 48 weeks; PROMISE-2, 24 weeks) [7, 8]. Benefits beyond reductions in migraine frequency were reported, including reductions in acute headache medication use and patient-reported improvements in functioning.

Two key secondary endpoints in both PROMISE studies were the percentage of patients achieving a ≥ 75% migraine responder rate (MRR) (i.e., reduction in MMDs) over Weeks 1–4 and Weeks 1–12 and a 50% MRR over Weeks 1–12. The proportion of patients who received eptinezumab 100 mg or 300 mg achieving ≥ 75% MRR at Week 12 was similar in both studies— ~ 26% in PROMISE-1 and ~ 30% in PROMISE-2—and was greater than that observed in the placebo groups (~ 16% in both studies). Similarly, the proportion of patients receiving either dosing scheme achieving a 50% response was ~ 53% (~ 16% difference from placebo) in PROMISE-1 and ~ 60% in PROMISE-2 (~ 20% difference from placebo). Whereas the clinical relevance of ≥ 75% MRR remains incompletely understood, it has been suggested previously that this threshold represents a “tipping point” in migraine prevention, with patients achieving ≥ 75% MRR in an eptinezumab study experiencing much greater improvements in patient-reported outcome measures than did patients with lower thresholds of response [9]. The objective of the present post hoc analysis of data from the two PROMISE studies was to measure the impact of the eptinezumab-induced ≥ 75% MRR on patient-reported outcomes including health-related quality of life (HRQoL) in PROMISE-1 and PROMISE-2 and compare it to the impact reported in patients experiencing a ≥ 50–< 75% MRR.

## Methods

### Data sources

The detailed methodology for PROMISE-1 (NCT02559895) [5] and PROMISE-2 (NCT02974153) [6] was published previously. Briefly, these studies were pivotal phase 3, parallel-group, randomized, double-blind, placebo-controlled trials that evaluated the preventive efficacy, tolerability, and safety of eptinezumab in adults with migraine. In both studies, patients received eptinezumab or placebo, administered intravenously (IV) over 30 min to 1 h every 12 weeks. In PROMISE-1, eptinezumab doses used were 30 mg, 100 mg, and 300 mg, while in PROMISE-2, eptinezumab doses used were 100 mg and 300 mg. For the purposes of this analysis, only patients receiving the approved 100 mg or 300 mg doses of eptinezumab were included; therefore, any “eptinezumab pooled” groups included those two dose levels. Response to the two doses was not significantly different in either study. Patients in PROMISE-1 received up to 4 doses of study medication and patients in PROMISE-2 received up to 2 doses. PROMISE-1 enrolled patients with episodic migraine (EM), and PROMISE-2 enrolled patients with chronic migraine (CM), with diagnostic assessment completed during the screening visit. A daily electronic diary (eDiary) was used throughout the 28-day screening period to confirm diagnosis and acute medication use at baseline. Patients who reported migraine or headache frequency outside the study inclusion criteria were considered protocol deviations but included in the analysis.

### Outcomes and assessment

A daily eDiary was used throughout each study to obtain a daily report (irrespective of headache occurrence), and to capture the incidence and characteristics of headache and migraine events, as well as use of acute headache medication. Headache data were entered into the eDiary as they occurred and, after the resolution of a headache, the patient answered questions about the headache that allowed it to be classified as a migraine or non-migraine headache. For both studies, the primary outcome measure was the reduction in MMDs over the first 12 weeks of the study; secondary outcome measures included the percentage of patients achieving ≥ 75% MRR over Weeks 1–4 and Weeks 1–12 and ≥ 50% MRR over Weeks 1–12. These reductions in MMDs were calculated as the difference between the number of migraine days recorded in the eDiary during the baseline period and the average monthly number of migraine days recorded over the treatment interval. A similar method of analysis was used to calculate monthly headache days (MHDs). Days of acute headache medication use—defined as days with any combination analgesic, simple analgesic, or triptan use—were measured during the baseline period, and the mean change over Weeks 1–12 was calculated.

Both PROMISE-1 and PROMISE-2 also captured HRQoL as measured by the 36-item Short-Form Health Survey (SF-36; v2.0) [10, 11] at scheduled visits. The SF-36 measures HRQoL over the preceding 4 weeks in 8 key domains (vitality, physical functioning, bodily pain, general health perceptions, physical role functioning, emotional role functioning, social functioning, and mental health), which are also combined into mental component summary and physical component summary scores. Norm-based scores below 50 represent health status that is below the nationally derived mean of the US population. Of interest are the bodily pain, social functioning, and physical role functioning (role-physical) domains, as these were the most impacted domains at baseline in the full study populations [6, 7, 12].

Patients in PROMISE-2 also completed the following patient-reported outcome measures during visits: 6-Item Headache Impact Test (HIT-6) [13, 14], patient-identified most bothersome symptom (PI-MBS), and Patient Global Impression of Change (PGIC) [15]. The HIT-6 was administered at screening, Day 0, and each study visit through Week 32. Scores of ≥60 denote severe headache-related life impact, 56–59 indicate substantial headache-related life impact, 50–55 represent some headache-related life impact, and ≤ 49 demonstrate little or no headache-related life impact. PI-MBS was identified at screening, where patients described the most bothersome symptom they associated with their chronic migraine; from this information, the investigator categorized the symptom as well as noted the verbatim symptom on the assessment form at each visit. At Weeks 4, 8, 12, 16, 20, 24, and 32, patients were asked to rate the overall change in that symptom since study initiation, using a 7-item Likert-type scale ranging from “very much improved” to “very much worse”. Patients completed the PGIC during the same scheduled visits as PI-MBS (excluding baseline), using an identical rating scale. The PGIC involves a single question about the patient’s impression of the overall change in their disease status since the start of the study and encompasses multiple domains of health including activity limitations, symptoms, emotions, and overall quality of life.

### Statistical analysis

This post hoc analysis included data from patients treated with eptinezumab 100 mg or 300 mg who were ≥ 75% or ≥ 50 –< 75% migraine responders over Weeks 1–12, meaning that they experienced a ≥ 75% or ≥ 50 –< 75% mean reduction from baseline in their MMD frequency over this period. Patients were analyzed within the treatment group to which they were randomly assigned.

For migraine endpoints based upon the eDiary (e.g., migraine days, headache days, etc.), missing data were imputed depending on patient compliance with the eDiary. If the eDiary was completed for ≥21 days in a 28-day study month, the observed frequency was normalized to 28 days. If the diary was completed for < 21 days, the results were a weighted function of the observed data for the current interval and the results for the previous interval, with the weight being proportional to the number of completed eDiary days. For acute headache medication use, if the eDiary was completed for ≥ 14 days in a 28-day study month, the observed frequency was normalized to 28 days; if the eDiary was completed for < 14 days, missing data were not imputed.

Descriptive statistics (including mean, standard deviation, and percentage) were used to report data; as this was a post hoc analysis, no formal tests for statistical significance were conducted. Analyses were performed using SAS software version 9.4 (SAS Institute, Inc., Cary, NC, USA).

## Results

### Patients

In PROMISE-1, a total of 115/443 (26.0%) eptinezumab-treated patients achieved ≥ 75% MRR over Weeks 1–12 (100 mg, *n* = 49; 300 mg, *n* = 66), and a total of 120/443 (27.1%) eptinezumab-treated patients achieved ≥ 50–< 75% MRR (100 mg, *n* = 61; 300 mg, *n* = 59). In PROMISE-2, a total of 211/706 (30.0%) eptinezumab-treated patients achieved ≥ 75% MRR over Weeks 1–12 (100 mg, *n* = 95; 300 mg, *n* = 116), and a total of 209/706 (29.6%) eptinezumab-treated patients achieved ≥ 50–< 75% MRR (100 mg, *n* = 110; 300 mg, *n* = 99). Demographic and baseline clinical characteristics of eptinezumab-treated patients with a ≥ 75% or a ≥ 50–< 75% MRR are presented in Table 1. In general, the dose groups were well matched with regard to baseline characteristics in each study. The mean age of all patients in this analysis was ~ 40 years, with the majority being female (567/655; 86.6%) and white (601/655; 91.8%).

**Table 1:** Baseline demographics and characteristics of eptinezumab-treated ≥ 50–< 75% and ≥ 75% migraine responders

	**Eptinezumab 100 mg**		**Eptinezumab 300 mg**		**Eptinezumab Pooled**	
**PROMISE-1 (EM)**	**≥ 50–< 75%** ***N*** **= 61**	**≥ 75%** ***N*** **= 49**	**≥ 50–< 75%** ***N*** **= 59**	**≥ 75%** ***N*** **= 66**	**≥ 50–< 75%** ***N*** **= 120**	**≥ 75%** ***N*** **= 115**
Mean age, years (SD)	40.5 (10.09)	39.1 (12.18)	40.1 (13.03)	40.1 (11.21)	40.3 (11.58)	39.7 (11.59)
Sex, n (%)						
Female	47 (77.0%)	39 (79.6%)	52 (88.1%)	57 (86.4%)	99 (82.5%)	96 (83.5%)
Male	14 (23.0%)	10 (20.4%)	7 (11.9%)	9 (13.6%)	21 (17.5%)	19 (16.5%)
Race, n (%)						
White	53 (86.9%)	43 (87.8%)	52 (88.1%)	58 (87.9%)	105 (87.5%)	101 (87.8%)
Black or African American	6 (9.8%)	2 (4.1%)	5 (8.5%)	7 (10.6%)	11 (9.2%)	9 (7.8%)
Other	2 (3.3%)	4 (8.2%)	2 (3.4%)	1 (1.5%)	4 (3.3%)	5 (4.3%)
Mean (SD) BMI, kg/m^2^	29.5 (6.56)	28.0 (7.48)	27.4 (6.59)	29.8 (7.28)	28.4 (6.63)	29.0 (7.39)
Mean (SD) age at diagnosis, years	23.4 (11.50)	19.9 (9.24)	22.7 (10.28)	21.0 (9.39)	23.1 (10.88)	20.5 (9.30)
Mean (SD) duration of migraine diagnosis, years	17.0 (12.16)	19.3 (10.99)	17.4 (13.10)	19.0 (11.22)	17.2 (12.58)	19.1 (11.07)
Mean (SD) baseline migraine days	8.3 (2.66)	8.8 (2.85)	9.0 (2.79)	8.5 (2.86)	8.7 (2.73)	8.6 (2.84)
Mean (SD) baseline headache days	9.2 (2.77)	10.0 (2.65)	10.0 (2.74)	10.4 (3.32)	9.6 (2.77)	10.3 (3.05)
**PROMISE-2 (CM)**	**≥ 50–< 75%** ***N*** **= 110**	**≥ 75%** ***N*** **= 95**	**≥ 50–< 75%** ***N*** **= 99**	**≥ 75%** ***N*** **= 116**	**≥ 50–< 75%** ***N*** **= 209**	**≥ 75%** ***N*** **= 211**
Mean age, years (SD)	38.3 (11.37)	43.9 (11.18)	42.1 (10.95)	41.1 (10.06)	40.1 (11.31)	42.3 (10.64)
Sex, n (%)						
Female	98 (89.1%)	80 (84.2%)	92 (92.9%)	102 (87.9%)	190 (90.9%)	182 (86.3%)
Male	38.3 (11.37)	15 (15.8%)	7 (7.1%)	14 (12.1%)	19 (9.1%)	29 (13.7%)
Race, n (%)						
White	106 (96.4%)	90 (94.7%)	91 (91.9%)	108 (93.1%)	197 (94.3%)	198 (93.8%)
Black or African American	4 (3.6%)	5 (5.3%)	6 (6.1%)	6 (5.2%)	10 (4.8%)	11 (5.2%)
Other	0	0	2 (2.0%)	2 (1.7%)	2 (1.0%)	2 (0.9%)
Mean (SD) BMI, kg/m^2^	25.4 (4.77)	26.3 (4.11)	25.9 (4.25)	26.5 (4.87)	25.6 (4.53)	26.4 (4.53)
Mean (SD) age at diagnosis, years	20.7 (9.39)	24.1 (10.13)	22.0 (9.52)	23.0 (9.43)	21.3 (9.46)	23.5 (9.74)
Mean (SD) duration of migraine diagnosis, years	17.7 (11.02)	19.7 (12.43)	20.1 (12.71)	18.1 (11.25)	18.8 (11.88)	18.8 (11.80)
Mean (SD) duration of chronic migraine, years	10.4 (11.19)	10.3 (12.30)	13.2 (12.38)	10.8 (11.01)	11.7 (11.82)	10.6 (11.58)
Mean (SD) baseline migraine days	16.1 (4.43)	15.7 (4.18)	15.5 (4.64)	15.0 (4.42)	15.8 (4.53)	15.3 (4.32)
Mean (SD) baseline headache days	20.2 (3.01)	19.6 (2.54)	19.8 (3.09)	20.0 (3.11)	20.0 (3.05)	19.8 (2.86)
Medication-overuse headache diagnosis, n (%)	46 (41.8%)	38 (40.0%)	47 (47.5%)	44 (37.9%)	93 (44.5%)	82 (38.9%)

A ≥ 50–< 75% migraine responder was defined as a patient who achieved a ≥ 50–< 75% reduction in mean monthly migraine days over Weeks 1–12. A ≥ 75% migraine responder was defined as a patient who achieved a ≥ 75% reduction in mean monthly migraine days over Weeks 1–12. BMI, body mass index; CM, chronic migraine; EM, episodic migraine; SD, standard deviation

### Maintenance and consistency of ≥ 75% and ≥ 50–< 75% migraine response

**Fig. 1:**
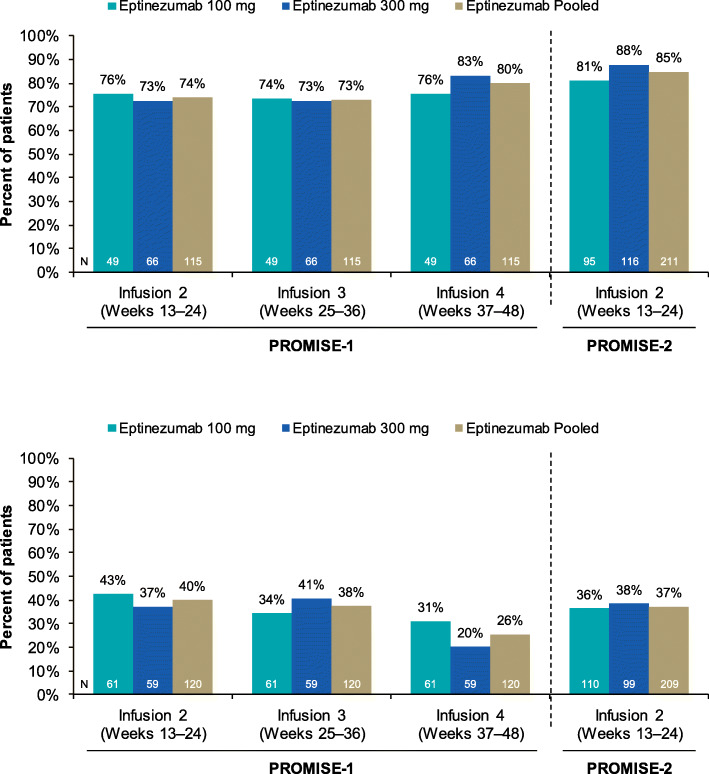
Maintenance of treatment response in Weeks 13–24 based on the migraine response rate in Weeks 1–12 in PROMISE-1 and PROMISE-2 (pooled): **(A)** Percentage of eptinezumab-treated patients maintaining ≥ 75% or ≥ 50–< 75% migraine response during the first infusion (Weeks 1–12) and **(B)** Percentage of eptinezumab-treated patients maintaining ≥ 50–< 75% migraine response maintaining response in Weeks 13–24

In PROMISE-1, > 70% of eptinezumab-treated patients with ≥ 75% MRR over Weeks 1–12 maintained that response in the 12 weeks after the second, third, and/or fourth infusions; for those with ≥ 50–< 75% MRR over Weeks 1–12, ~ 20–43% of eptinezumab-treated patients maintained that response and ~ 38–59% exceeded or improved to ≥ 75% MRR. In PROMISE-2, > 80% of eptinezumab-treated patients maintained ≥ 75% MRR in the 12 weeks after the second infusion. In addition, ~ 36–38% of eptinezumab patients with ≥ 50–< 75% MRR over Weeks 1–12 maintained that response and ~ 37–42% exceeded or improved to a ≥ 75% MRR (Fig. 1).

**Fig. 2:**
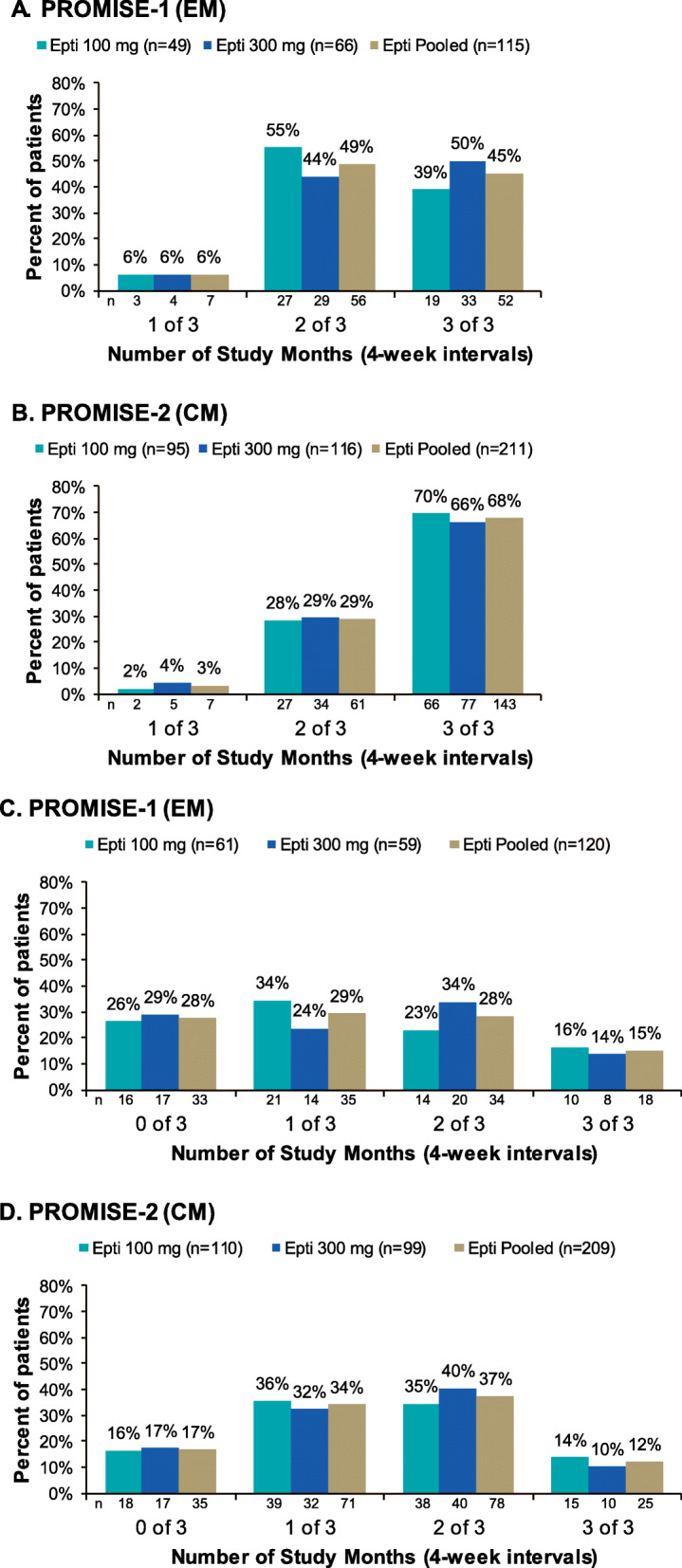
Number of study months eptinezumab-treated patients maintained same monthly migraine response achieved in Weeks 1–12. Percentage of ≥ 75% migraine responders in **(A)** PROMISE-1 and **(B)** PROMISE-2 and percentage of ≥ 50–< 75% migraine responders in **(C)** PROMISE-1 and **(D)** PROMISE-2. CM, chronic migraine; EM, episodic migraine; Epti, eptinezumab

For patients with ≥ 75% MRR over Weeks 1–12, most (312/326 [95.7%]) experienced ≥ 75% MRR for ≥2 of the 3 study months (i.e., 4-week intervals) (Fig. 2**;** Additional file 1) and nearly 60% (195/326 [59.8%]) maintaining a ≥ 75% MRR for all 3 study months of the first infusion interval (i.e., Weeks 1–12). For patients with ≥ 50–< 75% MRR over Weeks 1–12, 47.1% (155/329) maintaining a ≥ 50–< 75% MRR for at least 2 of the 3 study months, and 20.7% [68/329]) of patients with ≥ 50–< 75% MRR over Weeks 1–12 maintaining a ≥ 50–< 75% MRR less than 1 month out of the 3 study months.

### Changes in MMDs for eptinezumab-treated ≥ 75% and ≥ 50–< 75% migraine responders

In each study, baseline migraine frequency (MMDs) was similar across treatment groups, averaging 8.6 days in PROMISE-1 and 15.3 days in PROMISE-2 for ≥ 75% migraine responders, and 8.7 days in PROMISE-1 and 15.8 days in PROMISE-2 for ≥ 50–< 75% migraine responders (Table 1).

Whereas most (85.2% [98/115]) eptinezumab-treated ≥ 75% migraine responders in PROMISE-1 experienced 6–20 MMDs at baseline, none experienced > 4 MMDs over the 12 weeks following the initial dose and 8.7% (10/115) experienced no MMDs during Weeks 1–12. The mean (standard deviation) migraine frequency over Weeks 1–12 was 1.2 (0.84) MMDs. Most (86.7% [104/120]) eptinezumab-treated ≥50–< 75% migraine responders in PROMISE-1 also experienced 6–20 MMDs at baseline, 75.8% (91/120) experienced 1–4 MMDs and 24.2% (29/120) experienced > 4 MMDs during Weeks 1–12. The mean (standard deviation) migraine frequency over Weeks 1–12 was 3.3 (1.26) MMDs.

Most (86.3% [182/211]) eptinezumab-treated ≥ 75% migraine responders in PROMISE-2 experienced 9–20 MMDs, and 10.0% (21/211) experienced ≥21 MMDs at baseline; 95.2% (201/211) experienced 4 or fewer MMDs over the 12 weeks following the initial dose. Most (82.9% [175/211]) experienced only 1–4 and 12.3% (26/211) experienced no MMDs during this period. The mean (standard deviation) migraine frequency over Weeks 1–12 was 1.7 (1.32) MMDs. Most (80.4% [168/209]) eptinezumab-treated ≥ 50–< 75% migraine responders experienced 9–20 MMDs, 16.3% (34/209) experienced ≥21 MMDs, and 3.3% (7/209) experienced 6–8 MMDs at baseline. Over the 12 weeks following the initial dose, most (79.9% [167/120) patients experienced > 4 MMD, and 20.1% (42/209) experienced 2–4 MMD. The mean (standard deviation) migraine frequency over Weeks 1–12 was 6.1 (2.11) MMDs.

### Changes in MHDs for eptinezumab-treated ≥ 75% and ≥ 50–< 75% migraine responders

In each study, baseline headache frequency (MHDs) was similar across treatment groups, averaging 10.3 days in PROMISE-1 and 19.8 days in PROMISE-2 in eptinezumab-treated patients with ≥ 75% MRR, and 9.6 days in PROMISE-1 and 20.0 days in PROMISE-2 in eptinezumab-treated patients with ≥ 50–< 75% MRR (Table 1).

Most (60.0% [69/115]) ≥ 75% migraine responders in PROMISE-1 experienced 9–14 MHDs at baseline; 5.2% (6/115) experienced ≤5 MHDs, 25.2% (29/115) experienced 6–8 MHDs, 8.7% (10/115) experienced 15–20 MHDs, and 0.9% (1/115) experienced ≥21 MHDs. Over the 12 weeks following the initial dose, only 12.2% (14/115) experienced > 4 MHDs; most (82.6% [95/115]) experienced 1–4, and 5.2% (6/115) experienced no MHDs during this time period. Most (61.7% [74/120]) ≥ 50–< 75% migraine responders also experienced 9–14 MHDs at baseline; 9.2% (11/120) experienced ≤5 MHDs, 25.8% (31/120) experienced 6–8 MHDs, 3.3% (4/120) experienced 15–20 MHDs, and 0 experienced ≥21 MHDs. Over the 12 weeks following the initial dose, 50.0% (60/120) experienced > 4 MHDs; the other half (50.0% [60/120]) experienced 1–4 MHDs.

Whereas most (64.0% [135/211]) ≥ 75% migraine responders experienced 15–20 MHDs and 36.0% (76/211) experienced ≥21 MHDs at baseline, in PROMISE-2 more than 40% (41.7% [88/211]) experienced ≤4 MHDs over the 12 weeks following the initial dose. The remainder (58.3% [123/211]) experienced more than 4 MHDs during this time period. Most (59.8% [125/209]) ≥ 50–< 75% migraine responders in PROMISE-2 experienced 15–20 MHDs, 39.2% (82/209) experienced ≥21 MHDs, and 1.0% (2/209) experienced 9–14 MHDs at baseline. Most (97.6% [204/209]) experienced > 4 MHDs over the 12 weeks following the initial dose. The remainder (2.4% [5/209]) experienced 3–4 MHDs during this time period.

### Acute headache medication use and patient-reported outcomes in eptinezumab-treated ≥ 75% and ≥ 50–< 75% migraine responders

**Fig. 3:**
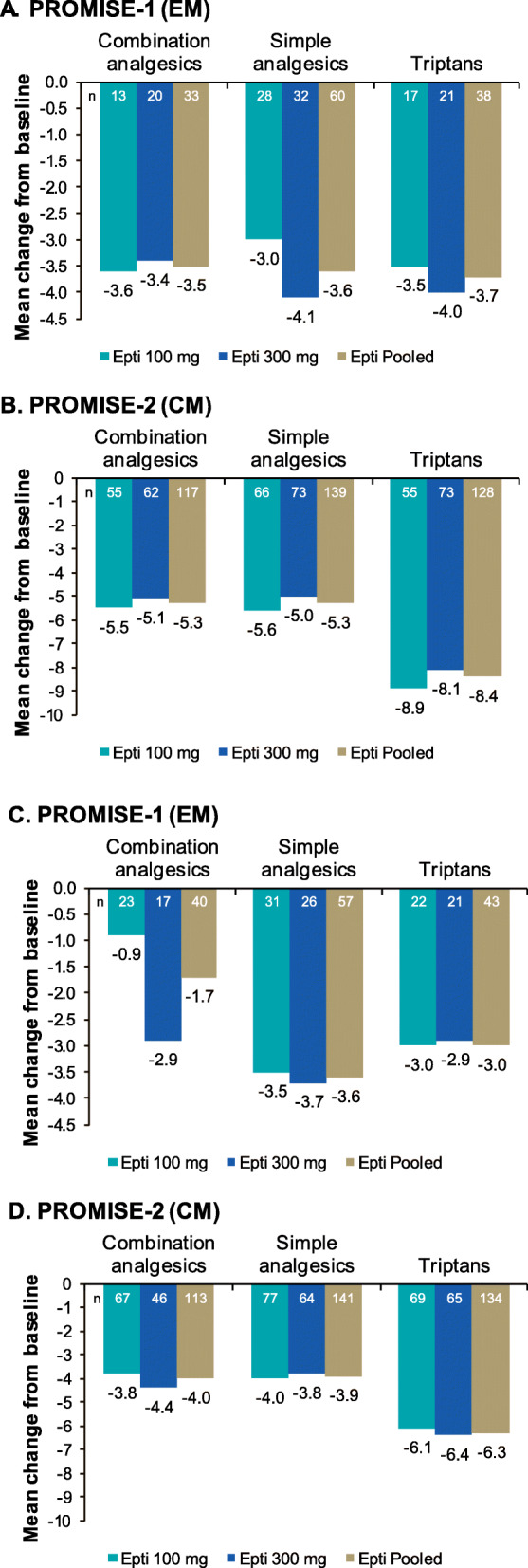
Changes in days of acute headache medication use over Weeks 1–12 in eptinezumab-treated ≥ 75% migraine responders in **(A)** PROMISE-1 and **(B)** PROMISE-2 and in ≥ 50–< 75% migraine responders in **(C)** PROMISE-1 and **(D)** PROMISE-2. Analyses for each medication are limited to patients who used that medication during the 28-day baseline period. A ≥ 75% or ≥ 50–< 75% migraine responder was defined as a patient who achieved a ≥ 75% or ≥ 50–< 75% reduction in mean monthly migraine days over Weeks 1–12. CM, chronic migraine; EM, episodic migraine; Epti, eptinezumab.

Eptinezumab-treated patients who experienced ≥ 75% or ≥ 50–< 75% MRR during Weeks 1–12 demonstrated reduced use of combination and simple analgesics, as well as triptans, during that same period (Fig. 3), with ≥ 75% responders experiencing, on average, fewer acute headache medication days. For ≥ 75% and ≥ 50–< 75% migraine responders in PROMISE-2, the greatest reduction in acute headache medication use was for triptans (− 8.4 and − 6.3 triptan days, respectively).

Eptinezumab-treated ≥ 75% and ≥ 50–< 75% migraine responders in PROMISE-1 (EM) generally reported scores of approximately 50 across the SF-36 domains of bodily pain, social functioning, and role-physical at baseline (Table 2). At Week 12, mean scores improved by 6.2 points for bodily pain, 2.6 points for social functioning, and 4.3 points for role-physical domains for ≥ 75% migraine responders, while scores improved by 5.9 points for bodily pain, 3.0 points for social functioning, and 2.3 points for role-physical domains for ≥ 50–< 75% migraine responders. Eptinezumab-treated ≥ 75% and ≥ 50–< 75% migraine responders in PROMISE-2 (CM) generally reported severely impacted HRQoL (SF-36 domain scores below 1–2 standard deviations below the mean) at baseline. At Week 12, SF-36 scores across the domains increased 9.8 points for bodily pain, 8.2 points for social functioning, and 7.9 points for role-physical domains, resulting in mean scores of ~ 50 for ≥ 75% migraine responders. At Week 12, SF-36 scores across the domains increased 5.6 points for bodily pain, 5.4 points for social functioning, and 6.0 points for role-physical domains for ≥ 50–< 75% migraine responders.

**Table 2:** Change from baseline to Week 12 in SF-36 bodily pain, social functioning, and role-physical domains in eptinezumab-treated ≥ 75% and ≥ 50–< 75% migraine responders

	**Eptinezumab 100 mg**		**Eptinezumab 300 mg**		**Eptinezumab Pooled**	
**PROMISE-1 (EM)**	**≥ 50–< 75%** ***N*** **= 61**	**≥ 75%** ***N*** **= 49**	**≥ 50–< 75%** ***N*** **= 59**	**≥ 75%** ***N*** **= 66**	**≥ 50–< 75%** ***N*** **= 120**	**≥ 75%** ***N*** **= 115**
**Bodily pain**						
Baseline, mean (SD)	45.8 (8.88)	46.2 (9.95)	46.5 (9.71)	48.5 (9.30)	46.1 (9.27)	47.5 (9.60)
Week 12, mean (SD)	51.2 (8.99)	52.7 (8.26)	52.5 (7.86)	54.7 (7.20)	51.8 (8.46)	53.9 (7.70)
Change from baseline, mean (SD)	5.8 (8.24)	6.7 (7.83)	6.1 (8.19)	5.9 (7.66)	5.9 (8.18)	6.2 (7.71)
**Social functioning**						
Baseline, mean (SD)	48.6 (9.37)	50.6 (8.22)	50.4 (8.98)	51.4 (8.06)	49.5 (9.19)	51.1 (8.10)
Week 12, mean (SD)	51.5 (8.97)	53.9 (5.08)	52.8 (6.97)	53.9 (6.26)	52.1 (8.07)	53.9 (5.77)
Change from baseline, mean (SD)	3.3 (9.96)	3.2 (7.49)	2.6 (8.26)	2.2 (7.14)	3.0 (9.15)	2.6 (7.28)
**Role-physical**						
Baseline, mean (SD)	49.8 (7.61)	49.1 (8.90)	49.7 (7.75)	50.1 (7.91)	49.8 (7.64)	49.7 (8.32)
Week 12, mean (SD)	51.2 (6.92)	53.8 (6.57)	52.8 (5.71)	54.2 (5.04)	52.0 (6.39)	54.0 (5.72)
Change from baseline, mean (SD)	1.5 (7.86)	4.7 (7.91)	3.1 (6.56)	3.9 (7.50)	2.3 (7.28)	4.3 (7.65)
**PROMISE-2 (CM)**	**≥ 50–< 75%** ***N*** **= 110**	**≥ 75%** ***N*** **= 95**	**≥ 50–< 75%** ***N*** **= 99**	**≥ 75%** ***N*** **= 116**	**≥ 50–< 75%** ***N*** **= 209**	**≥ 75%** ***N*** **= 211**
**Bodily pain**						
Baseline, mean (SD)	40.6 (10.25)	40.1 (9.60)	40.5 (8.97)	40.1 (9.40)	40.5 (9.64)	40.1 (9.47)
Week 12, mean (SD)	45.8 (8.81)	49.2 (8.14)	46.8 (7.80)	50.2 (7.57)	46.3 (8.35)	49.7 (7.82)
Change from baseline, mean (SD)	5.1 (8.22)	9.4 (9.66)	6.2 (8.37)	10.1 (9.21)	5.6 (8.29)	9.8 (9.39)
**Social functioning**						
Baseline, mean (SD)	43.7 (9.36)	42.6 (10.82)	44.0 (10.05)	43.0 (9.65)	43.8 (9.67)	42.8 (10.17)
Week 12, mean (SD)	49.1 (7.42)	51.1 (6.83)	49.0 (7.48)	51.1 (7.42)	49.1 (7.43)	51.1 (7.15)
Change from baseline, mean (SD)	5.3 (7.41)	8.4 (9.50)	5.5 (7.68)	8.0 (9.14)	5.4 (7.52)	8.2 (9.28)
**Role-physical**						
Baseline, mean (SD)	42.5 (8.52)	42.3 (9.04)	42.4 (9.15)	42.2 (8.72)	42.4 (8.80)	42.2 (8.84)
Week 12, mean (SD)	48.1 (7.61)	49.4 (6.79)	48.7 (7.51)	50.7 (6.93)	48.4 (7.55)	50.1 (6.88)
Change from baseline, mean (SD)	5.5 (7.05)	7.1 (9.74)	6.5 (7.74)	8.6 (8.67)	6.0 (7.38)	7.9 (9.17)

A ≥ 75% or ≥ 50–< 75% migraine responder was defined as a patient who achieved a ≥ 75% or ≥ 50–< 75% reduction in mean monthly migraine days over Weeks 1–12. CM, chronic migraine; EM, episodic migraine; SD, standard deviation; SF-36, 36-item Short-Form Health Survey (v2.0)

**Fig. 4:**
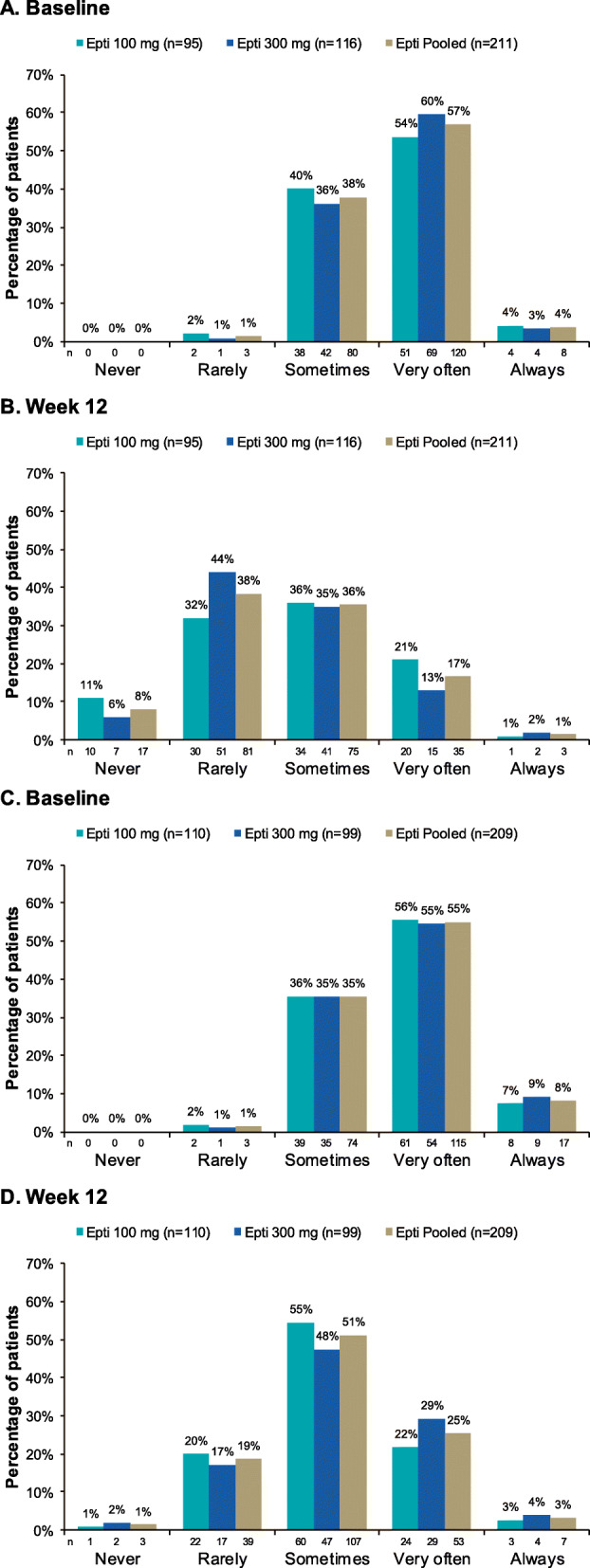
Responses to HIT-6 item 1 (severe pain) in eptinezumab-treated patients maintaining ≥ 75% migraine response over Weeks 1–12 in PROMISE-2 at **(A)** baseline and **(B)** Week 12 and those maintaining ≥ 50–< 75% migraine response over Weeks 1–12 in PROMISE-2 at **(C)** baseline and **(D)** Week 12. Item 1 of the 6-item Headache Impact Test (HIT-6) asked “When you have headaches, how often is the pain severe?” A ≥ 75% or ≥ 50–< 75% migraine responder was defined as a patient who achieved a ≥ 75% or ≥ 50–< 75% reduction in mean monthly migraine days over Weeks 1–12. Epti, eptinezumab

HIT-6 data from PROMISE-2 indicate that eptinezumab-treated patients who experienced a ≥ 75% MRR during Weeks 1–12 had reduced HIT-6 total scores at Week 12, with the mean (SD) change from baseline being − 11.7 (8.19) in the combined eptinezumab groups (*N* = 211). The magnitude of the observed change is nearly double that which is believed to be clinically meaningful in patients with migraine (i.e., change of − 5) or chronic migraine (i.e., change of − 6) [16, 17]. The frequency of severe headache pain (HIT-6 item 1) was lower at Week 12 than at baseline in eptinezumab-treated ≥ 75% migraine responders, with only 18.0% (38/211) reporting that headache pain was very often or always severe at Week 12, compared to 60.7% (128/211) at baseline (Fig. 4). In addition, HIT-6 total scores at Week 12 indicated that headache had little-to-no life impact (score ≤ 49) in more than one-third (36.0% [76/211]) of eptinezumab-treated ≥ 75% migraine responders. Eptinezumab-treated patients who experienced ≥ 50–< 75% MRR during Weeks 1–12 also had reduced HIT-6 total scores at Week 12, with the mean (SD) change from baseline being − 7.6 (6.08) in the combined eptinezumab groups (*N* = 209). The frequency of severe headache pain (HIT-6 item 1) was lower at Week 12 than at baseline in eptinezumab-treated ≥ 50–< 75% migraine responders, with only 28.7% (60/209) reporting that headache pain was very often or always severe at Week 12, compared to 63.2% (132/209) at baseline (Fig. 4). HIT-6 total scores at Week 12 indicated that headache only had little-to-no life impact (score ≤ 49) in 10.0% (21/209) of patients, with 45.5% (95/209) of patients indicating that headache had severe life impact (score ≥ 60).

Changes in PI-MBS and PGIC in eptinezumab-treated ≥ 75% and ≥ 50–< 75% migraine responders in PROMISE-2 are summarized in Table 3. A total of 87/208 (41.8%) eptinezumab-treated ≥ 75% migraine responders reported that their PI-MBS was “very much improved” at Week 12. Similarly, 75/207 (36.2%) eptinezumab-treated ≥ 75% MRR patients described their condition as “very much improved” on the PGIC at Week 12. In eptinezumab-treated ≥ 50–< 75% migraine responders, a total of 33/200 (16.5%) reported that their PI-MBS was “very much improved” at Week 12, and a total of 40/200 (20.0%) described their condition as “very much improved” on the PGIC at Week 12.

**Table 3:** PI-MBS and PGIC ratings at Week 12 in eptinezumab-treated ≥ 75% and ≥ 50–< 75% migraine responders (PROMISE-2; eptinezumab pooled)

	PI-MBS		PGIC	
	≥ 50–< 75%	≥ 75%	≥ 50–< 75%	≥ 75%
n	200	208	200	207
Very much improved	33 (16.5%)	87 (41.8%)	40 (20.0%)	75 (36.2%)
Much improved	101 (50.5%)	90 (43.3%)	99 (49.5%)	103 (49.8%)
Minimally improved	46 (23.0%)	21 (10.1%)	40 (20.0%)	16 (7.7%)
No change	14 (7.0%)	5 (2.4%)	17 (8.5%)	10 (4.8%)
Minimally worse	5 (2.5%)	5 (2.4%)	2 (1.0%)	3 (1.4%)
Much worse	1 (0.5%)	0	2 (1.0%)	0

A ≥ 75% or ≥ 50–< 75% migraine responder was defined as a patient who achieved a ≥ 75% or ≥ 50–< 75% reduction in mean monthly migraine days over Weeks 1–12. PGIC, Patient Global Impression of Change; PI-MBS, patient-identified most bothersome symptom

## Discussion

The results of this post hoc analysis provide evidence of the benefits beyond reduction in MMD that are associated with an MRR of ≥ 75%, and suggest that monitoring reductions in migraine frequency, in concert with simple questioning about overall status, PI-MBS, and problems related to functioning, provides extensive evidence of the benefits of treatment. For patients with EM and CM, mean monthly migraine frequency was < 2 MMDs when experiencing ≥ 75% MRR with eptinezumab, while patients experiencing ≥ 50–< 75% MRR with eptinezumab had monthly migraine frequencies of 3.3 MMDs in patients with EM and 6.1 MMDs in patients with CM. Benefits associated with ≥ 75% MRR in this analysis included reductions in acute medication use and improvements in patient-reported outcome measures designed to assess headache impact, status of PI-MBS, overall disease status, and HRQoL. While patients with ≥ 50–< 75% MRR experienced reductions in acute medication use and improvements in patient-reported outcome measures, changes were not as substantial as those with ≥ 75% MRR. Changes in HIT-6 scores indicate that eptinezumab-treated ≥ 75% migraine responders recognized a reduction in daily headache-related impact and pain severity, and those in SF-36 domain scores captured increases to normative levels for bodily pain, social functioning, and physical role functioning. Changes in HIT-6 scores indicate that eptinezumab-treated ≥ 50–< 75% migraine responders still experience clinically meaningful changes in HIT-6 scores both for episodic migraine (i.e., change of − 5) and chronic migraine (i.e., change of − 6) [16, 17], albeit a lower reduction in migraine frequency than exhibited in ≥ 75% migraine responders (− 7.6 versus − 11.7). Changes in PGIC scores provided further evidence of the improvement in patients’ perception of their condition in those patients experiencing ≥ 75% MRR. Fewer eptinezumab-treated patients experiencing ≥ 50–< 75% MRR indicated “very much improved” on the PGIC, and more reported “no change” or “minimally improved” than those experiencing ≥ 75% MRR. As the ≥ 75% migraine responder rate was largely sustained after repeated dosing in both studies (80% through the fourth infusion [48 weeks] in PROMISE-1 and 85% through the second infusion [24 weeks] in PROMISE-2), it is conceivable that improvements in HRQoL would persist long-term as well. Previously, HIT-6 analysis of PROMISE-2 showed improvements of HIT-6 were sustained up to 8 months, well beyond the duration of the study [18]. Comparatively, 20–43% maintained but 38–59% exceeded the ≥ 50–< 75% MRR through the fourth infusion [48 weeks] in PROMISE-1, with 36–38% maintaining and 37–42% improving through the second infusion [24 weeks] in PROMISE-2. These data suggest that the lower frequency of the ≥ 50– > 75% MRR may be due to patients improving to the ≥ 75% MRR.

It is notable that the high rate of achievement of ≥ 75% MRR with eptinezumab in PROMISE-1 (26.0% of patients who received eptinezumab 100 mg or 300 mg) was accomplished with monotherapy. Monotherapy is desirable since it may limit some of the untoward effects that could occur with the use of multiple preventive treatments and agrees with current recommendations for migraine prevention [19, 20].

As this was a post hoc analysis, the limitations of which are widely recognized, additional prospective studies are needed to confirm all findings.

## Conclusion

In this post hoc analysis of data from PROMISE-1 and PROMISE-2, the attainment of ≥ 75% MRR was associated with substantial improvements in patient-reported headache-related life impact, patient perception of disease status, and patients’ self-identified most bothersome migraine-associated symptom, as well as a reduction in use of acute headache medication, which were more marked than those with ≥ 50- < 75% MRR. This study supports the clinical meaningfulness of ≥ 75% MRR for patients with either episodic or chronic migraine.

## Supplementary Information

**Additional file 1.** Table of monthly ≥ 75% or ≥ 50–< 75% migraine response during Weeks 1–12 in eptinezumab-treated ≥ 75% or ≥ 50–< 75% migraine responders. A ≥ 75% or ≥ 50–< 75% migraine responder was defined as a patient who achieved a ≥ 75% or ≥ 50–< 75% reduction in mean monthly migraine days over Weeks 1–12. CM, chronic migraine; EM, episodic migraine.

## Data Availability

In accordance with EFPIA’s and PhRMA’s “Principles for Responsible Clinical Trial Data Sharing” guidelines, Lundbeck is committed to responsible sharing of clinical trial data in a manner that is consistent with safeguarding the privacy of patients, respecting the integrity of national regulatory systems, and protecting the intellectual property of the sponsor. The protection of intellectual property ensures continued research and innovation in the pharmaceutical industry. Deidentified data are available to those whose request has been reviewed and approved through an application submitted to https://www.lundbeck.com/global/our-science/clinical-data-sharing.

## Abbreviations

CGRP: Calcitonin gene-related peptide
CM: Chronic migraine
EM: Episodic migraine
HIT-6: 6-Item Headache Impact Test
HRQoL: Health-related quality of life
IgG1: Immunoglobulin G1
IV: Intravenously
MHDs: Monthly headache days
MMDs: Monthly migraine days
PGIC: Patient Global Impression of Change
PI-MBS: Patient-identified most bothersome symptom
SF-36: 36-item Short-Form Health Survey
