# Supplementary material for: Patient-reported outcomes, health-related quality of life, and acute medication use in patients with a ≥ 75% response to eptinezumab: subgroup pooled analysis of the PROMISE trials
Source: J Headache Pain. 2022 Feb 7;23(1):23. doi: 10.1186/s10194-022-01386-z (PMC8903490; doi:10.1186/s10194-022-01386-z)
Supplement: Supplementary file 1 — Additional file 1. Table of monthly ≥ 75% or ≥ 50–< 75% migraine response during Weeks 1–12 in eptinezumab-treated ≥ 75% or ≥ 50–< 75% migraine responders. A ≥ 75% or ≥ 50–< 75% migraine responder was defined as a patient who achieved a ≥ 75% or ≥ 50–< 75% reduction in mean monthly migraine days over Weeks 1–12. CM, chronic migraine; EM, episodic migraine. [file 10194_2022_1386_MOESM1_ESM.docx]

**SUPPLEMENTARY INFORMATION**

**Additional file 1. Table of monthly ≥75% migraine response during Weeks 1–12 in eptinezumab-treated ≥75% migraine responders**

|  | **Eptinezumab  100 mg** | **Eptinezumab  300 mg** | **Eptinezumab Pooled** |
| --- | --- | --- | --- |
| **Cumulative number of 4-week intervals with ≥75% migraine response** | | | |
| PROMISE-1 (EM), n | 49 | 66 | 115 |
| 3 of 3  2 of 3  1 of 3 | 19 (38.8%)  27 (55.1%)  3 (6.1%) | 33 (50.0%)  29 (43.9%)  4 (6.1%) | 52 (45.2%)  56 (48.7%)  7 (6.1%) |
| PROMISE-2 (CM), n | 95 | 116 | 211 |
| 3 of 3  2 of 3  1 of 3 | 66 (69.5%)  27 (28.4%)  2 (2.1%) | 77 (66.4%)  34 (29.3%)  5 (4.3%) | 143 (67.8%)  61 (28.9%)  7 (3.3%) |

A ≥75% migraine responder was defined as a patient who achieved a ≥75% reduction in mean monthly migraine days over Weeks 1–12. CM, chronic migraine; EM, episodic migraine.
